# 3α-Hydroxybufadienolides in *Bufo* gallbladders: structural insights and biotransformation

**DOI:** 10.1007/s13659-024-00442-2

**Published:** 2024-03-04

**Authors:** Li-Jun Ruan, Zhi-Jun Song, Ren-Wang Jiang

**Affiliations:** 1 State Key Laboratory of Bioactive Molecules and Draggability Assessment, College of Pharmacy, Jinan University, Guangzhou, 510632 China; 2 National Engineering Research Center for Southwest Endangered Medicinal Materials Resources Development, Guangxi Botanical Garden of Medicinal Plants, Nanning, 530023 China

**Keywords:** Bufadienolides, *Bufo gargarizans*, Toad bile, Self-defense, Biotransformation

## Abstract

Bufadienolides, naturally occurring steroids primarily found in toads, have garnered attention for their pharmacological properties and ecological significance. In this study, we isolated and identified 21 bufadienolides from the gallbladders of *Bufo gargarizans*, comprising four new compounds and 17 known ones. Notably, the predominance of 15 bufadienolides with a 3α-OH configuration in toad bile differs significantly from the 3β-OH bufadienolides found in venom secreted by toad glands. Moreover, our investigation into the biotransformation of 3β-OH and 3α-OH bufadienolides in the liver and kidney tissues of toads revealed an irreversible conversion from 3β-OH to 3α-OH bufadienolides, suggesting a crucial role in toad self-detoxification. These findings provide valuable insights into the structural diversity of bufadienolides and advance our understanding of their medical and ecological significance.

**Graphical Abstract:**

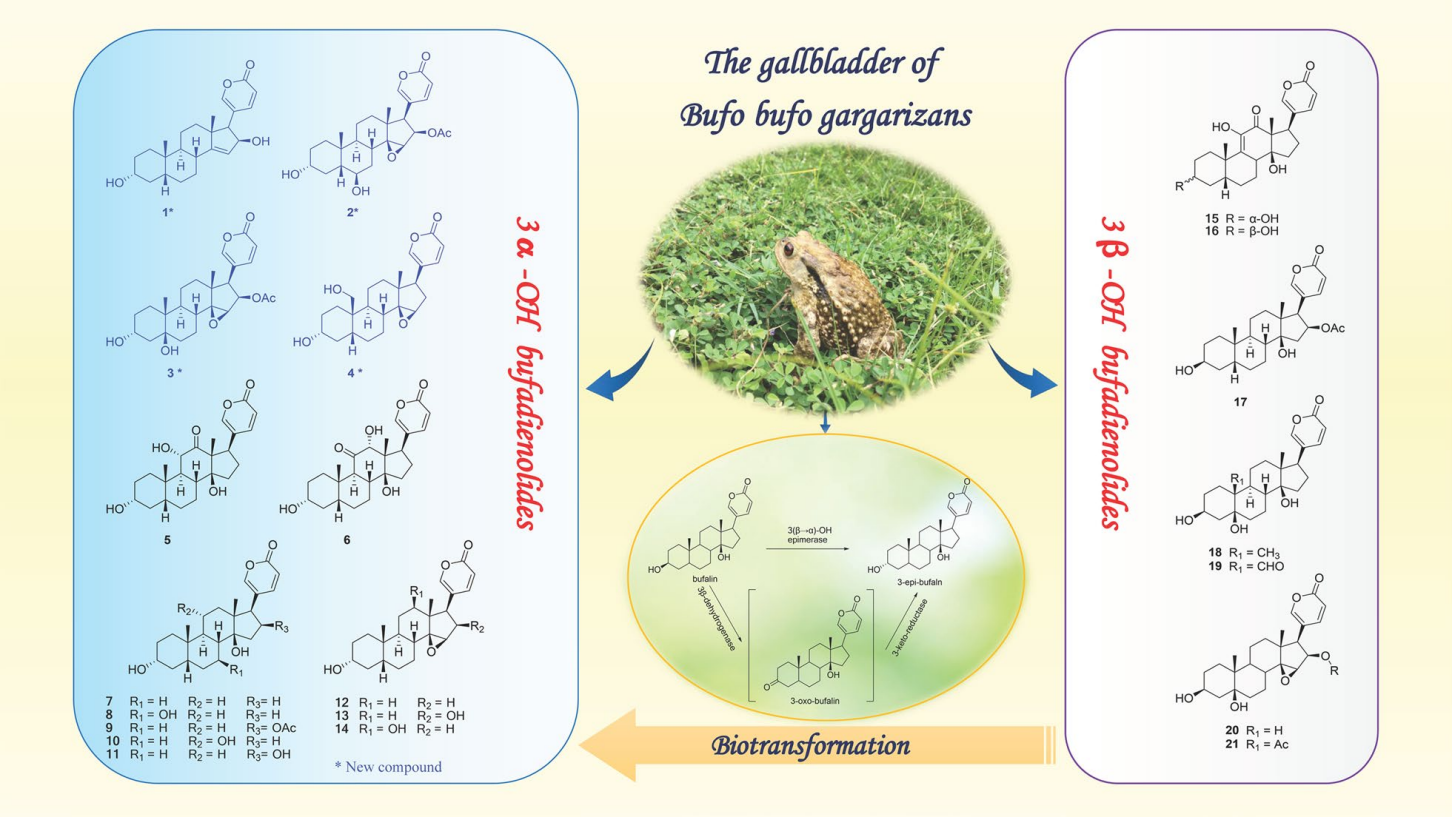

**Supplementary Information:**

The online version contains supplementary material available at 10.1007/s13659-024-00442-2.

## Introduction

Bufadienolides are widely used in traditional medicine for the treatment of various diseases such as infections, rheumatism and inflammation, especially in the treatment of heart failure and cancer due to their potent inhibition of Na^+^, K^+^-ATPase [1, 2]. These compounds feature a C24 steroid skeleton with an α-pyrone (hexagonal unsaturated lactone ring) at the C-17 position, and can be present as aglycones or hydroxyl substituted derivatization at the C-3 position. They are extensively distributed in animals of the *Bufo* genus [3], particularly in their venom [4], a secretion from skin and parotoid glands [5, 6]. Intriguingly, bufadienolides have also been discovered in various internal organs of toads, such as the gallbladder [7, 8], blood [9–11], ovaries [8, 12] and eggs [13, 14]. In particular, bufadienolides within the internal tissues of toads exhibit significant structural differences compared to those found in the skin and parotoid glands. The parotoid glands and skin primarily secrete bufadienolides with a 3β-OH configuration, whereas internal organs mostly contain 3α-hydroxybufadienolides.

The precise structural variations of bufadienolides within the internal organs of toads and their biological significance remain elusive. Several studies have proposed a potential correlation between bufadienolides and the control of Na^+^-K^+^ ATPase activity, impacting the metabolism of water-salt and the regulation of osmosis in toads [11]. Some researchers have proposed that bufadienolides in internal organs, especially those with 3α-OH derivatives, may exhibit lower toxicity compared to the 3β-OH bufadienolides found in glands and skin [15], potentially contributing to the toad's self-detoxification process [16]. Additionally, there are also hypotheses proposing that the unique bufadienolides in toad internal organs may serve as precursors for the synthesis of bufadienolides in glands and skin, or might be metabolic by-products of bufadienolides in glands and skin [17].

To gain a deeper insight into the structure of bufadienolides in the internal organs of *Bufo bufo gargarizans*, we conducted a comprehensive investigation focusing on bufadienolides in the gallbladder. In a previous study, we reported the discovery of an unprecedented 5/7/6/5/5/6 ring spirostanol bufospirostenin A [18], as well as a series of bile acids [17]. As part of our ongoing efforts to explore the chemical diversity and physiological mechanisms in toads, we continued to perform comprehensive isolation, structural elucidation, and biotransformation of these bufadienolides, to provide the potential ecological and pharmacological significance in the fields of medicine and ecology.

**Fig. 1:**
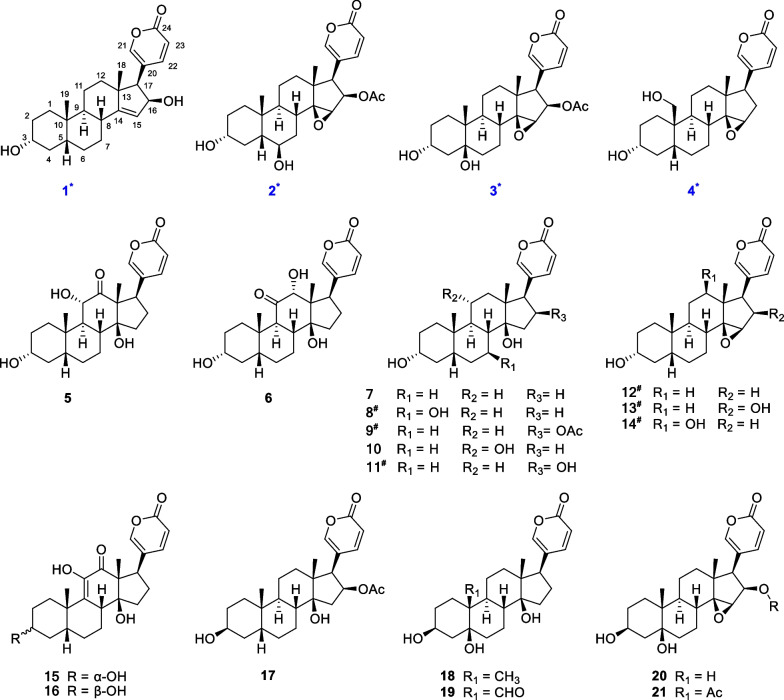
Chemical structures of the compounds **1–21** isolated from the bile of *Bufo gargarizan* (* new compounds; ^#^ isolation from *Bufo* genus for the first time)

In the present study, we isolated 21 bufadienolides (Fig. 1) from the gallbladder extract of *Bufo gargarizans* using preparative HPLC with a detection at approximately 296 nm. These compounds included four new and 17 known compounds, and their structures were determined through a combination of techniques such as NMR, MS, and single-crystal X-ray diffraction. The investigation revealed the intriguing coexistence of 15 bufadienolides with a 3α-OH configuration and six structures with a 3β-OH configuration. Then, we further explored the biotransformation of bufadienolides by incubating toad liver and kidney tissues with 3α-OH and 3β-OH bufalin in vitro. We hypothesize the existence of a 3(β → α)-OH epimerase, which catalyzes the irreversible conversion of bufalin to 3*-epi-*bufalin through an intermediate 3*-oxo-*bufalin. This study enhances our understanding of the structural diversity of bufadienolides and provides insights into their potential ecological and pharmacological significance.

## Result and discussion

### Structure Elucidation

A total of 21 bufadienolides (Fig. 1) was isolated from toad bile and identified by a comprehensive analysis of spectroscopic data, including high-resolution MS (HR-ESI–MS), NMR, UV, and single-crystal X-ray diffraction data. Among them, compounds **1–4** were previously undescribed, and compounds **8**, **9** and **11–14** were isolated for the first time from the genus *Bufo*. According to the configuration of the hydroxyl group at the C-3 position, we categorized these compounds into 3α-OH and 3β-OH bufadienolides. 15 of them are 3α-OH bufadienolides (**1–15**) and six of them are 3β-OH bufadienolides (**16–21**). It was noteworthy that a considerable quantity of 3α-OH bufadienolides was identified in toad bile, which differed from the reports of 3β-OH bufadienolides found in toad venom and skin [1, 4, 6]. The unique presence of these 3α-OH bufadienolides in toad bile added a new dimension to our understanding of the chemical diversity in different toad organs and opened new insights for pharmacological investigations, biosynthesis and ecological roles.

**Fig. 2:**
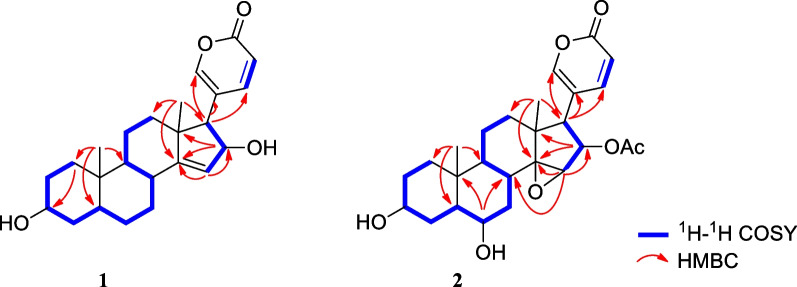
Key ^1^H–^1^H–COSY (blue bold), HMBC (red arrow) correlations of **1** and **2**

**3-*epi*-Bufoliene** (**1**), a colorless crystal, had a molecular formula of C_24_H_32_O_4_ (nine degrees of unsaturation), determined by positive HR-ESI–MS at *m/z* 385.2382 [M + H]^+^ (calcd. for C_24_H_33_O_4_, 385.2373). The ^1^H and ^13^C NMR spectroscopic data (Tables 1 and 2) suggest that **1** had a bufadienolide steroidal structure with a 2*H*-pyran-2-one or α-pyrone ring system, supported by the UV absorption at λ_max_ 305 nm. The presence of this characteristic α-pyrone ring was evidenced from the ^1^H NMR data at *δ*_H_ 6.29 (1H, dd, *J* = 9.2, 0.9 Hz, H-23), 7.69 (1H, dd, *J* = 2.5, 0.9 Hz, H-21), and 7.85 (1H, dd, *J* = 9.2, 2.5 Hz, H-22) and from the ^13^C NMR spectrum at *δ*_C_ 119.3 (C-20), 152.3 (C-21), 150.7 (C-22), 114.5 (C-23) and 164.7 (C-24). In addition, the NMR data assignment of the tetracyclic steroidal nucleus was carried out by comparing it with the reported bufadienolides [14, 15] and by analyzing the ^1^H-^1^H COSY, HSQC, and HMBC data (Fig. 2). Singlet methyl signals at *δ*_H_ 1.03 (*δ*_*C*_ 23.0) and *δ*_H_ 0.98 (*δ*_C_ 23.6) are typical of steroidal methyl groups and were designated as C-18 and C-19, respectively. These designations are based on their HMBC correlations, with H_3_-18 (*δ*_H_ 1.03) correlating to C-12 (*δ*_C_ 41.7), C-13 (*δ*_C_ 49.5), C-14 (*δ*_C_ 161.4), and C-17 (*δ*_C_ 58.1), and H_3_-19 (*δ*_H_ 0.98) correlating to C-1 (*δ*_C_ 36.3), C-5 (*δ*_C_ 43.3), C-9 (*δ*_C_ 42.5), and C-10 (*δ*_C_ 36.0).

**Table 1:** ^1^H NMR data of **1–4** in CD_3_OD (300 MHz, *J* in Hz, *δ* in ppm)

NO	**1**	**2**	**3**	**4**
1α	1.86	1.80	1.59	1.74
β	1.06	1.10	1.27	1.50
2α	1.28	1.31	1.31	1.32
β	1.64	1.65	1.68	1.71
3β	3.55, m	3.50, m	3.94, m	3.52, m
4α	1.51	1.39	1.98	1.51
β	1.71	1.60	1.56	1.68
5β	1.44	1.58	\	1.85
6α	1.40	\	1.40	1.25
β	2.01	3.73, br. s	1.75	1.80
7α	1.61	1.33	1.62	1.10
β	1.74	1.70	1.67	1.61
8	2.27, m	2.44, td (12.5, 3.5)	2.09	2.03
9	1.51	1.77	1.84	1.82
11α	1.56	1.59	1.61	1.56
11β	1.25	1.43	1.37	1.32
12α	1.23	1.56	1.57	1.46
β	1.84	1.83	1.82	1.70
15	5.50, t (2.4)	3.75, br s	3.76, br. s	3.63, br. s
16α	4.60, dd (5.9, 2.4)	5.50, dd (9.3, 1.3)	5.48, dd (9.3, 1.4)	2.44, m
β				2.04, m
17	2.57, d (5.9)	2.94, d (9.3)	2.95, d (9.3)	2.59, d (9.9)
18	1.03, s	0.83, s	0.92, s	0.76, s
19	0.98, s	1.12, s	0.82, s	3.82 and 3.42, d (11.2)
21	7.69, dd (2.5, 0.9)	7.38, d (1.3)	7.37, s	7.45, d (2.5)
22	7.85, dd (9.2, 2.5)	8.03, d (9.7, 1.3)	8.03, d (9.7)	7.90, dd (9.8, 2.5)
23	6.29, dd (9.2, 0.9)	6.25, d (9.7)	6.25, d (9.7)	6.27, d (9.8)
16-COCH_3_		1.85, s	1.86, s	

Overlapped signals were reported without designating multiplicity

Hydroxyl groups are prone to proton exchange or hydrogen bonding, the proton signals of hydroxyl groups are often absent from ^1^H-NMR spectra, and thus all the signals of hydroxyl groups in compounds **1**–**4** do not appear in the ^1^H-NMR spectra

**Table 2:** ^13^C NMR data of **1–4** in CD_3_OD (75 MHz,* δ* in ppm)

NO	**1**	**2**	**3**	**4**
1	36.3	36.7	30.6	29.3
2	31.3	30.9	30.8	31.0
3	72.3	71.8	68.4	72.0
4	37.0	37.1	42.4	36.8
5	43.3	48.7	75.6	34.9
6	27.9	73.2	36.2	27.0
7	25.4	29.5	24.2	21.6
8	36.9	29.6	33.8	35.1
9	42.5	41.3	43.8	40.9
10	36.0	35.9	41.0	40.1
11	22.1	21.6	22.2	22.1
12	41.7	40.5	40.7	40.5
13	49.5	46.3	46.2	46.3
14	161.4	73.3	73.5	75.9
15	121.3	60.7	60.8	61.1
16	77.4	76.6	76.5	33.2
17	58.1	51.4	51.2	48.5
18	23.0	17.4	16.6	17.1
19	23.6	26.0	17.4	64.9
20	119.3	118.4	118.4	124.9
21	152.3	153.5	153.5	151.8
22	150.7	150.9	150.9	149.6
23	114.5	114.1	114.1	115.4
24	164.7	164.1	164.0	164.5
16-COCH_3_		171.6	171.6	
16-COCH_3_		20.4	20.4

**Fig. 3:**
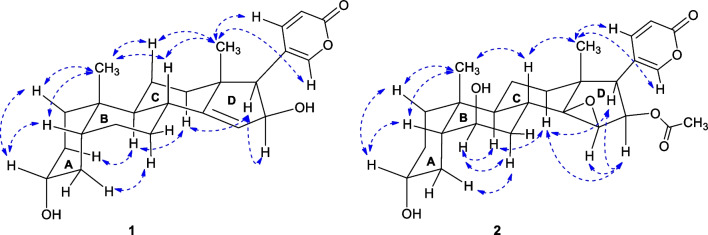
Key NOE correlations of **1** and **2** (A/B *trans*, B/C *cis* and C/D *cis*)

The presence of a hydroxyl group in ring A was indicated by the ^1^H and ^13^C NMR signals ([*δ*_H-3_ 3.55 (m), *δ*_C-3_ 72.3]. The location of this hydroxyl group at C-3 was determined by the ^1^H-^1^H COSY correlations of *δ*_H_ 3.55 (H-3) ↔ 1.28 and 1.64 (H_2_-2) and *δ*_H_ 3.55 (H-3) ↔ 1.51 and 1.71 (H_2_-4) and confirmed by the HMBC correlations from H_2_-1 to C-3. The α-orientation of the hydroxyl group was suggested by the width of the entire signal (24 Hz) because the β-oriented proton H-3, adopting the axial position, was split by two axial protons (H-2α and H-4α) and two equatorial proton (H-2β), and thus the width of *J* values should be much larger than the α-oriented proton H-3 [18]. Furthermore, the chemical shift of β-oriented proton H-3 (< 4.0 ppm) was normally smaller than the α-oriented proton H-3 (> 4.0 ppm) [19] because the former took the axial position in contrast to the equatorial position of the latter. In addition, α-orientation of the hydroxyl group was further confirmed by the NOE correlations H-3β ↔ H-1β and H-3β ↔ H-5 (Fig. 3). Similarly, the presence of another hydroxyl group in ring D was also indicated by the ^1^H and ^13^C NMR signals ([*δ*_H-16_ 4.60, dd (*J* = 5.9, 2.4 Hz), *δ*_C-16_ 77.4]. The location of this hydroxyl group at C-16 was determined by the ^1^H-^1^H COSY correlations of *δ*_H_ 5.50 (H-15) ↔ 4.60 (H-16) ↔ 2.57 (H-17) and confirmed by the HMBC correlations from H-16 to C-13 and C-14. The β-orientation of this hydroxyl group was suggested by the NOE correlation H-16α ↔ H-17 which was α-orientated. By analyzing the ^1^H-^1^H COSY correlations of *δ*_H_ 5.50 (H-15) ↔ 4.60 (H-16), as well as the long-range correlations from H-16 to and C-14, and from H-15 to C-14 and C-16 in HMBC spectrum, it was determined that ring D contained the trisubstituted vinyl group between C-14 and C-15. The other 2D-NMR data were similar to the reported bufadienolides [19]. Finally, the structure of **1** was established and defined as (3α,5β,16β)-3,16-dihydroxybufa-14,20,22-trienolide, and 3*-epi-*bufoliene was suggested as a trivial name.

**3-*epi-*6β-Hydroxycinobufagin** (**2**) was isolated as a white powder, and its molecular formula, C_26_H_34_O_7_, was determined from its HR-ESI–MS data (*m/z* 459.2408 [M + H]^+^, calcd. for C_26_H_35_O_7,_ 459.2377). Comparing the NMR data (Tables 1 and 2) with the literature values for cinobufagin [20] indicated that compound **2** had the same C_24_ bufadienolide framework with an acetoxy group at C-16, and an epoxy group at C-14 and C-15. The primary difference in **2** was the presence of an additional hydroxyl at C-6 and the epimerization of 3-OH. The hydroxyl group at C-6 in **2** was deduced from the ^1^H-^1^H COSY correlations of *δ*_H_ 1.58 (H-5) ↔ 3.73 (H-6) ↔ 1.33 (H-7α), and the HMBC correlations from *δ*_H_ 3.73 (H-6) to *δ*_C_ 29.6 (C-8) and 35.9 (C-10). Additionally, the steric configuration of 3α-OH was essentially identical to that of compound **1**, based on the small chemical shift of proton signal of H-3 at *δ*_H_ 3.50 with a large width of *J* values (21 Hz). It was further supported by NOE correlation of H-3/H-1β (*δ*_H_ 1.10) and H-3/H-5. The α-orientation of H-6 was confirmed by the NOE correlations of H-6/H-7α (*δ*_H_ 1.64) and H-6/H-9 (*δ*_H_ 1.82). Therefore, the structure of **2** was established as 3*-epi-*6β-hydroxycinobufagin.

**3-*epi-*Cinobufotalin** (**3**), a white powder, was assigned the molecular formula C_26_H_34_O_7_ as determined by the positive HR-ESI–MS ion at *m/z* HR-ESI–MS *m/z* 459.2407 [M + H]^+^ (calcd. for C_26_H_35_O_7_, 459.2377) with ten degrees of unsaturation. The UV data, with a *λ*_max_ of 295 nm, was consistent with bufadienolide skeleton. The NMR data of **3** were similar to those of **2** except that the hydroxyl group was at C-5 rather than C-6. This was further confirmed by HMBC cross peak from H_3_-19 (*δ*_H_ 0.92, s, 3H) to C-5 (*δ*_C_ 75.6). Furthermore, this data showed a high similarity to cinobufotalin (**21**) [21], with the main difference being that the proton signal at *δ*_H_ 3.94 (large width of multiplet) in **3** shifted downfield to *δ*_H_ 4.13 (broad singlet) in cinobufotalin (**21**), suggesting an inversion of the C-3 stereocenter, which was consistent with the H-3 proton signatures of other isolated 3α-OH bufadienolides. The ^1^H and ^13^C NMR signals were assigned as shown in Tables 1 and 2, respectively. Consequently, compound **3** was identified as 3*-epi-*cinobufotalin.

**3-*epi*-19-Hydroxyresibufogenin** (**4**), a white powder, was assigned the molecular formula C_24_H_32_O_5_ as determined by the positive HR-ESI–MS ion peak at *m/z* 423.2159 [M + Na]^+^ (calcd. for C_24_H_32_O_5_Na, 423.2142) with nine degrees of unsaturation. The ^1^H NMR and ^13^C NMR data (Tables 1 and 2) of compound **4** displayed resemblances to those of the known compound 3*-epi-*resibufogenin (**12**) [22], except for the replacement of the substitution of the methyl group in C-10 with a hydroxymethyl group [*δ*_H-19_ 3.82 and 3.42 (d, *J* = 11.2 Hz); *δ*_C-19_ 64.9] in **4**. The NOE correlations and relative configurations in **4** and **12** exhibited a high degree of similarity. The β-orientation of the hydroxymethyl in **4** was ascertained through the NOE correlations of H_2_-19/H-5 and H_2_-19/H-8. The H-3 signal at *δ*_H_ 3.52 featured with a large width of multiplet, along with the NOE correlation of H-3/H-1β and H-3/H-5, validating the 3α-OH configuration. Consequently, the arrangement of** 4** was determined to be 3*-epi-*19-hydroxyresibufogenin.

**Fig. 4:**
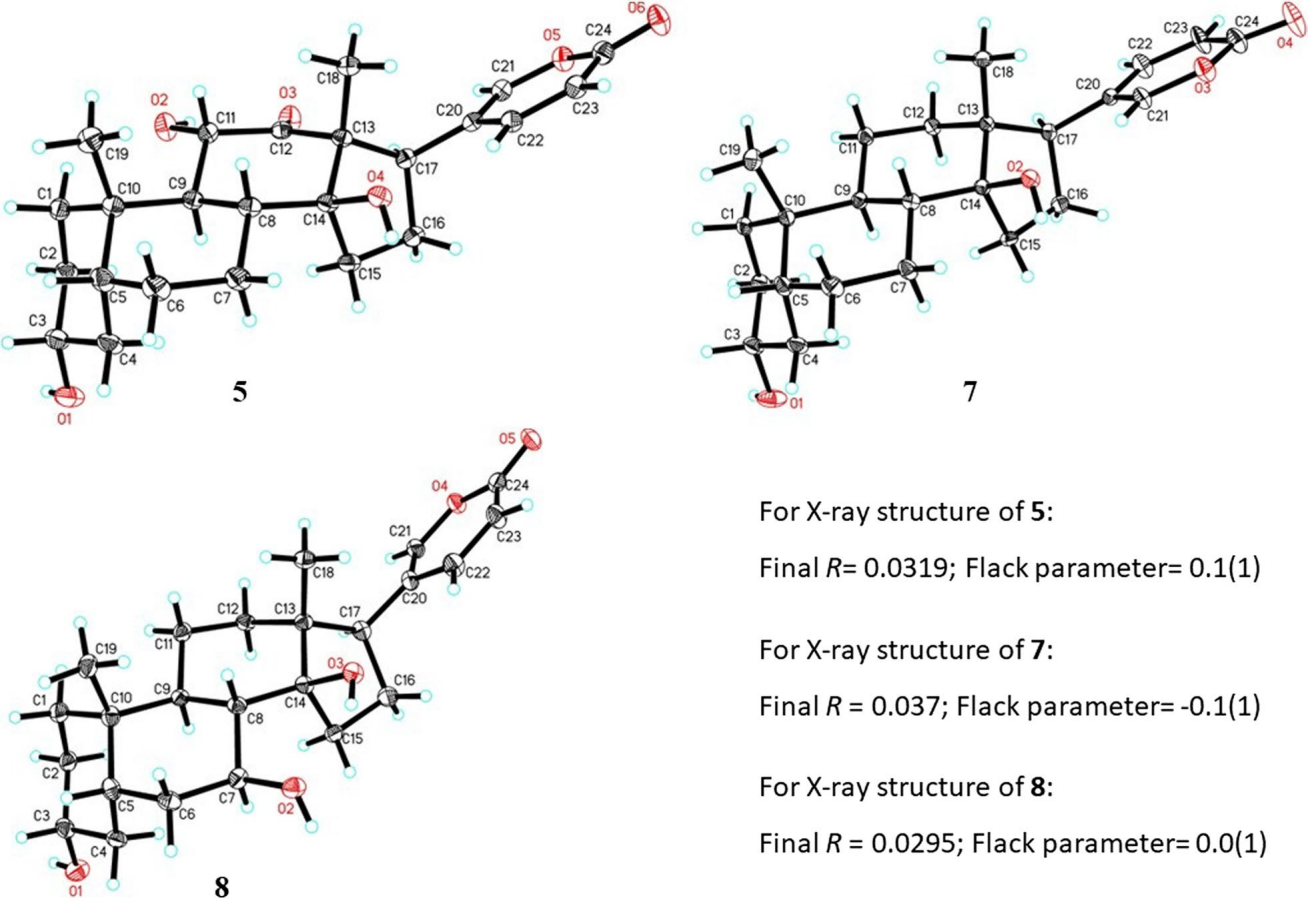
X-ray crystallographic structures of **5**, **7** and **8** with atom labeling scheme. The C and O atoms are drawn as 30% thermal ellipsoids

Additionally, 17 known compounds were identified (Fig. 1) as 3*-epi-*arenobufagin (**5**) [23], 3*-epi-*ψ-bufarenogin (**6**) [23], 3*-epi-*bufalin (**7**) [22], 3*-epi-*7β-hydroxybufalin (**8**) [24], 3*-epi-*bufotalin (**9**) [25], 3*-epi-*gambufotalin (**10**) [25], 3*-epi-*desacetylbufotalin (**11**) [25], 3*-epi-*resibufogenin (**12**) [22], 3*-epi-*desacetylcinobufagin (**13**) [22], 3*-epi-*12β-hydroxyresibufogenin (**14**) [26], 3*-epi-*argentinogenin (**15**) [5], argentinogenin (**16**) [5], bufotalin [20] (**17**), telocinobufagin (**18**) [27], hellebrigenin (**19**) [28], desacetylcinobufotalin (**20**) [20] and cinobufotalin (**21**) [29] by comparison of the NMR and MS data with the reported values in literatures. Among them, compounds **8**, **9**, **11–14** were isolated from Bufo genus for the first time (Fig. 1). In addition, the structures of compounds **5**, **7**, and **8** were confirmed by X-ray analysis for the first time (Fig. 4).

### Transformation relationships of 3α-OH and 3β-OH bufadienolides

This study revealed the coexistence of 3α-OH and 3β-OH bufadienolides in the bile of toad, with the 3α-OH configuration being predominant. To explore the potential conversion of 3α-OH and 3β-OH bufadienolides, a tissue incubation approach was employed to investigate the metabolic processes in toad liver and kidney tissues. The outcomes provide valuable insights, elucidating the transformation from 3β-OH to 3α-OH bufadienolides.

**Fig. 5:**
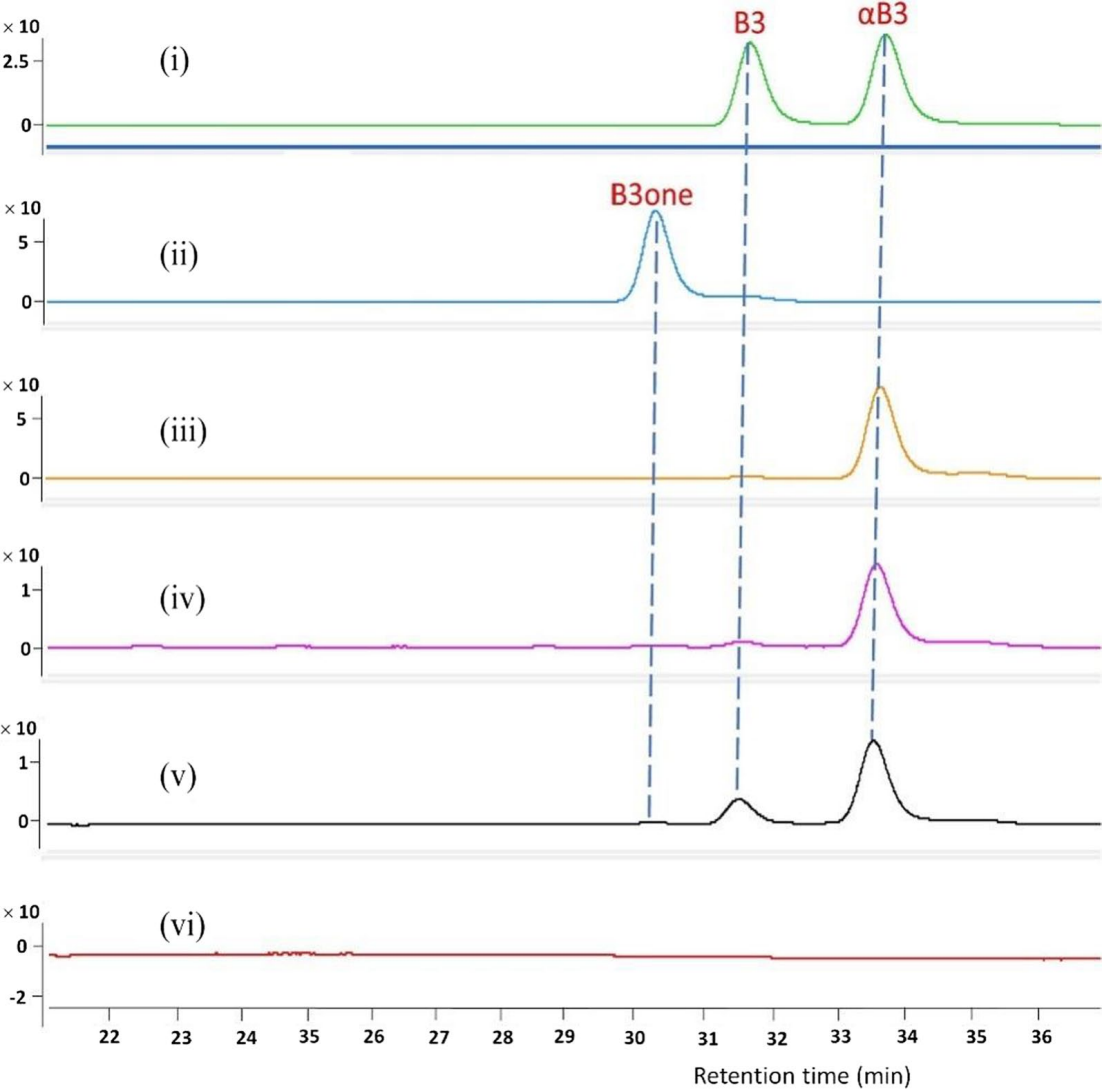
The HPLC chromatogram of bufalin (B3), 3-*epi*-bufalin (αB3), and 3-*oxo*-bufalin (B3one) after incubated with toad liver suspension for 24 h. (i) and (ii), standards of B3, αB3 and B3one; (iii) incubation of αB3 with toad liver; (iv) incubation of B3one with toad liver; (v) incubation of B3 with toad liver; (vi) the control group of toad liver. Detailed sample preparation method and HPLC method were shown in Sects. 4.6 and 4.7, respectively

**Fig. 6:**
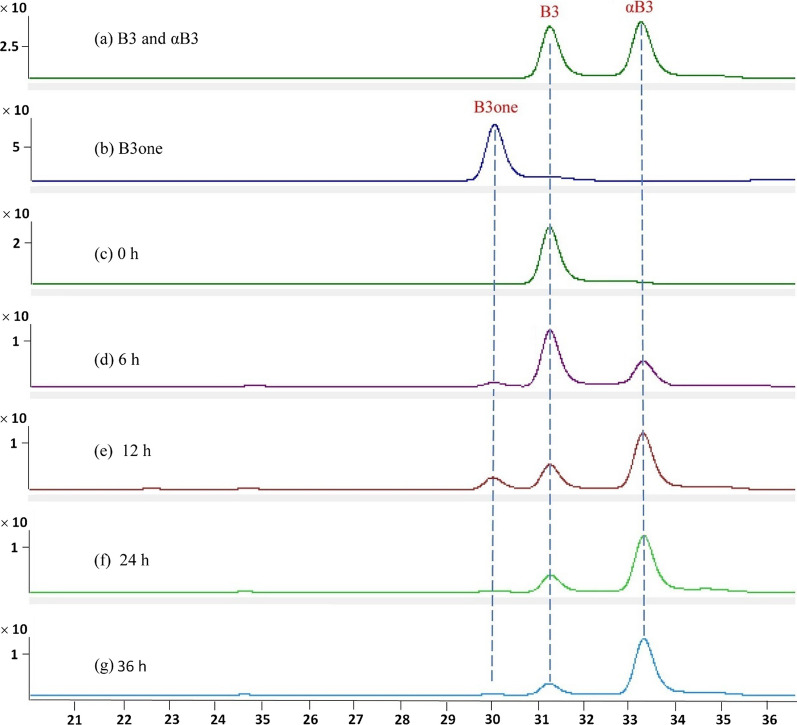
The HPLC chromatogram of bufalin (B3) after incubated with toad liver suspension for different time. (i) and (ii), standards of B3, αB3 (3-*epi*-bufalin), and B3one (3-*oxo*-bufalin); (c) ~ (g) B3 incubation with toad liver for 0, 6, 12, 24, 36 h, respectively. Detailed sample preparation method and HPLC method were shown in Sects. 4.6 and 4.7, respectively

In Fig. 5, the HPLC chromatograms showed three compounds (bufalin, 3*-epi-*bufalin, 3*-oxo-*bufalin) after a 24-h incubation with toad liver tissue culture. The incubation demonstrated a significant conversion of bufalin and 3*-oxo-*bufalin into 3*-epi-*bufalin. However, 3*-epi-*bufalin did not convert back into bufalin or 3*-oxo-*bufalin. Further examination of bufalin within liver tissue at different incubation time (Fig. 6) showed that the conversion of bufalin to 3*-epi-*bufalin increased with extended incubation time, whereas 3*-oxo-*bufalin initially increased and then declined. After 12-h of incubation, bufalin, 3*-oxo-*bufalin, and 3*-epi-*bufalin were simultaneously present. This finding strongly suggested that bufalin could undergo conversion to 3*-epi-*bufalin through an intermediate 3*-oxo-*bufalin, and this conversion process appears to be irreversible. It was worth noting that similar results were observed in the kidney tissue of toads (see Additional file 1).

**Fig. 7:**
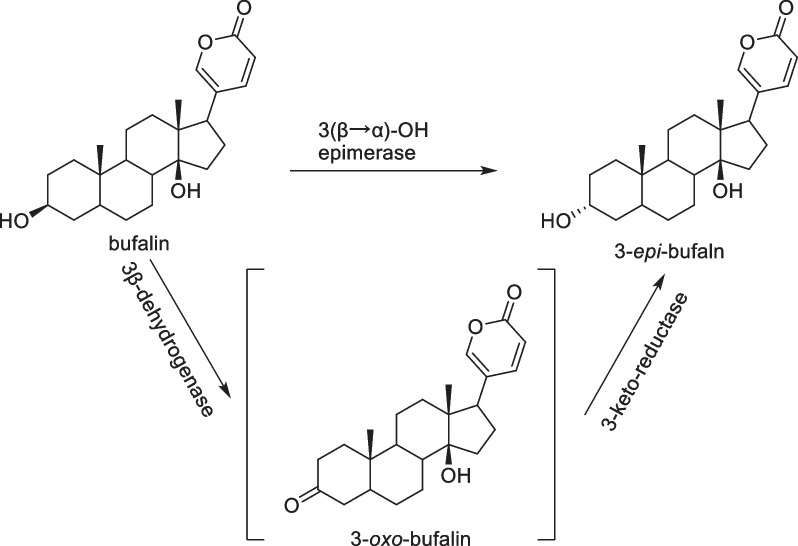
A schematic depicting the conversion pathway of bufalin into 3*-epi-*bufalin facilitated by the 3(β → α)-OH epimerase. This reaction involves two key enzymatic processes: a differential stereo-selective 3β-dehydrogenase that transforms bufalin into 3*-oxo-*bufalin and a 3-keto-reductase that subsequently converts 3*-oxo-*bufalin into 3*-epi-*bufalin

It was reasonable to postulate the presence of a group of 3(β → α)-OH epimerase in toad liver and kidney tissues [30, 31]. This epimerase comprised two key enzymatic activities (Fig. 7): a differential stereo-selective 3β-dehydrogenase, which converted bufalin into 3*-oxo-*bufalin, and a 3-keto-reductase that facilitated the transformation of 3*-oxo-*bufalin into 3*-epi-*bufalin. This process followed an irreversible conversion pathway: 3β-OH → 3-oxo → 3α-OH. We proposed that this enzyme was widely distributed in toad tissues and likely played a pivotal role in converting highly toxic 3β-OH bufadienolides into the less toxic 3α-OH counterparts. This assumption was supported by our previous studies [15], which demonstrated that 3β-OH bufadienolides had significantly higher inhibitory activity against Na^+^,K^+^-ATPase-α1 than 3α-OH bufadienolides. Furthermore, it was worth noting that predators of toads, such as rats [32] and snakes [33], could also convert 3β-OH bufadienolides into 3α-OH bufadienolides, thus neutralizing their toxicity. Hence, we proposed that this epimerase might play a role in the toad's self-defense mechanisms and was linked to crucial physiological functions. This irreversible enzymatic conversion process provided a new perspective on the ecological importance of bufadienolides in toad defense mechanisms, emphasizing the need for further research.

## Conclusions

This study presented the isolation and identification of 21 bufadienolides from the gallbladder of *Bufo gargarizans*, featuring four previously undescribed compounds along with 17 known ones, through various spectroscopic techniques including NMR, HR-ESI-MS in combination with X-ray diffraction. Notably, the coexistence of fifteen 3α-OH and six 3β-OH bufadienolides in toad bile, with a marked predominance of the 3α-OH configuration, added a new dimension to our understanding of chemical diversity within toad organs. Furthermore, the observation of irreversible conversion from 3β-OH to 3α-OH bufadienolides in toad liver and kidney tissues incubation offered new perspectives on the ecological significance of bufadienolides in toad defense mechanisms.

## Experimental

### General experimental procedures

Optical rotations, IR, UV, NMR spectra, HRESIMS, HPLC, and TLC, were carried out according to previously described procedures (Additional file 1) [18].

### Biological material

The gallbladders of toads were collected from Dongcheng Restaurant [17], and the live toad were purchased in the Shipai vegetable market in Guangdong province of China. All tissues and animals were authenticated as *Bufo gargarizans* Cantor by Prof. Pang-Chui Shaw (The Chinese University of Hong Kong, Hong Kong, P. R. China) using DNA technologies. They were sacrificed according to a procedure approved by the Animal Ethics Committee of Jinan University (No. 20130729001) and in accordance with the National Institutes of Health’s Guide for the Care and Use of Laboratory Animals (seventh edition).

### Extraction and isolation

The gallbladders (2.1 kg wet weight) were extracted with 95% ethanol three times (3 × 10 L) under ultrasonic conditions. The combined ethanol extracts were filtered and concentrated under reduced pressure to afford a crude extract (209 g), which was then suspended in water and partitioned with cyclohexane, ethyl acetate (EtOAc), and *n*-butanol (*n*-Bu), successively. The EtOAc soluble fraction (14 g) was subjected to a silica gel column chromatography (200–300 mesh) with a gradient elution of dichloromethane-methanol (CH_2_Cl_2_-CH_3_OH, from pure CH_2_Cl_2_, 100:1, 80:1, 40:1, 20:1, 10:1, 5:1, 2:1, 1:1 and pure methanol v/v) to yield ten fractions (Fr. A to J) [17]. The HPLC analysis revealed a significant presence of bufadienolides in A, B, and C, as indicated by the peak UV absorption at λ_max_ 296 nm. Consequently, for further purification, Fr. A, B, and C were repeatedly subjected to column chromatography on silica gel and semi-HPLC. Fr. A yielded compounds **1** (2.3 mg), **5** (6.1 mg), **6** (6.5 mg), **7** (12.3 mg), **8** (4.2 mg), **9** (1.7 mg), **15** (1.3 mg), **17** (2.0 mg), **19** (1.4 mg), **20** (0.7 mg), and **21** (1.1 mg). Fr. B afforded compounds **2** (4.9 mg), **3** (0.9 mg),** 4** (1.4 mg), **11** (0.5 mg), **12** (3.9 mg), **13** (4.8 mg), **14** (3.2 mg), **16** (0.6 mg), and **18** (2.0 mg). Fr. C afforded compound **10** (2.6 mg).

### Spectroscopic data

3*-epi-*Bufoliene (**1**): White powder (CH_3_OH); [*α*]_D_^25^ + 71 (*c* 0.10, CH_3_OH); UV (CH_3_OH) *λ*_max_ 305 nm, HR-ESI–MS *m/z* 385.2382 [M + H]^+^ (calcd. for C_24_H_33_O_4_, 385.2373); for ^1^H and ^13^C NMR (CD_3_OD) data, see Tables 1 and 2.

3*-epi-*6β-Hydroxycinobufagin (**2**): White powder (CH_3_OH); [*α*]_D_^25^   + 26 (*c* 0.10, CH_3_OH); UV (CH_3_OH) *λ*_max_ 295 nm, HR-ESI–MS *m/z* 459.2408 [M + H]^+^ (calcd. for C_26_H_35_O_7,_ 459.2377); for ^1^H and ^13^C NMR (CD_3_OD) data, see Tables 1 and 2.

3*-epi-*Cinobufotalin (**3**): White powder (CH_3_OH); [*α*]_D_^25^ + 19 (*c* 0.10, CH_3_OH); UV (CH_3_OH) *λ*_max_ 295nm, HR-ESI–MS *m/z* 459.2407 [M + H]^+^ (calcd. for C_26_H_35_O_7_, 459.2377); for ^1^H and ^13^C NMR (CD_3_OD) data, see Tables 1 and 2.

3*-epi-*19-Hydroxyresibufogenin (**4**): White powder (CH_3_OH); [*α*]_D_^25^ + 25 (*c* 0.10, CH_3_OH); UV (CH_3_OH) *λ*_max_ 299 nm, HR-ESI–MS *m/z* 423.2159 [M + Na]^+^ (calcd. for C_24_H_32_O_5_Na, 423.2142); for ^1^H and ^13^C NMR (CD_3_OD) data, see Tables 1 and 2.

### X-ray crystallographic analysis

Compounds **5**, **7** and **8** were crystallized from CH_3_OH at room temperature. The diffraction data collection, structural elucidation and refinement were performed using the same method as reported [34]. The X-ray structures of **5**, **7** and **8** were shown in Fig. 4. All non-hydrogen atoms were given anisotropic thermal parameters. H-atoms bonded to carbons were placed at geometrically ideal positions using the riding model. H-atoms bonded to oxygen were located using difference Fourier mapping and were included in the calculation of structural factors and isotropic temperature factors. The weighted *R* factor, *wR* and goodness-of-fit (*S*) values were obtained based on* F*^2^. The positions of hydrogen atoms were fixed geometrically at the calculated distances and allowed to ride on their parent atoms. Crystallographic data for the structures determined in this study have been deposited at the Cambridge Crystallographic Data Centre (CCDC 2305248, 2305249 and 2305250) and can be obtained free of charge from the CCDC Web site (https://www.ccdc.cam.ac.uk/).

3*-epi-*Arenobufagin (**5**): X-ray analysis: colourless blocks, C_24_H_32_O_6_ (*M* = 416.50), monoclinic, space group *P*2_1_; *α* = 7.4484 (3) Å, *b* = 15.3289 (7) Å, *c* = 8.9774 (4) Å; *α* = *γ* = 90.00°, *β* = 91.143 (3)°; *V* = 1024.80 (8) Å^3^; *T* = 173 (2) K; *Z* = 2, *ρ*_calc_ = 1.350 mg/mm^3^;* F* (000) = 448; Absorption coefficient 0.781 mm^-1;* θ* range for data collection: 4.93 to 62.66°; Final *R* indices [*I* ≥ 2*σ* (*I*)]: *R*_1_ = 0.0319, _*W*_*R*_2_ = 0.0805, Flack parameter (CuK*α*) = 0.1 (1).

3*-epi-*Bufalin (**7**): X-ray analysis: colourless blocks, C_24_H_34_O_4_ (*M* = 386.51), Orthorhombic, space group *P*2_1_2_1_2_1_; *α* = 7.2136 (3) Å, *b* = 14.9838 (5) Å, *c* = 18.7389 (8) Å; *α* = *γ* = *β* = 90°; *V* = 2025.43 (14) Å^3^; *T* = 173 (2) K; *Z* = 4, *ρ*_calc_ = 1.268 mg/mm^3^;* F* (000) = 840; Absorption coefficient 0.671 mm^-1;* θ* range for data collection: 3.78 to 62.72°; Final *R* indices [*I* ≥ 2*σ* (*I*)]: *R*_1_ = 0.037, _*W*_*R*_2_ = 0.0935, Flack parameter (CuK*α*) = -0.1 (1).

3*-epi-*7β-Hydroxybufalin (**8**): X-ray analysis: colourless blocks, C_24_H_34_O_5_ (*M* = 402.51), Orthorhombic, space group *P*2_1_2_1_2_1_; *α* = 6.5605 (2) Å, *b* = 15.1956 (4) Å, *c* = 21.5384 (6) Å; *α* = *γ* = *β* = 90°; *V* = 2147.18 (11) Å^3^; *T* = 173 (2) K; *Z* = 4, *ρ*_calc_ = 1.245 mg/mm^3^;* F* (000) = 872; Absorption coefficient 0.689 mm^-1;* θ* range for data collection: 4.10 to 62.70°; Final *R* indices [*I* ≥ 2*σ* (*I*)]: *R*_1_ = 0.0295, _*W*_*R*_2_ = 0.0720, Flack parameter (CuK*α*) = 0.0 (1).

### Biotransformation procedure assay

Toads were euthanized under ether anesthesia, and their hearts were subsequently perfused with Ringer's saline (6.5 g of sodium chloride, 0.2 g of sodium bicarbonate, 0.14 g of potassium chloride, 0.01 g of sodium dihydrogen phosphate, and 0.12 g of calcium chloride dissolved in 1000 mL of water). Under aseptic conditions, the liver and kidney tissues were excised, chilled, and homogenized in a cold environment. Next, 0.40 g of tissue homogenate was placed in 10 mL centrifuge tubes, and 4 mL DMEM containing 0.4 mM NADPH was added. The experimental group containing the tissue homogenates was added 4 μL of either bufalin, 3*-epi-*bufalin, or 3*-oxo-*bufalin solution (50 mM), whereas the control group without the tissue homogenates was added 4 μL of bufalin, 3*-epi-*bufalin, or 3*-oxo-*bufalin solution (50 mM) along with 4 mL of DMEM (containing 0.4 mM NADPH). All groups were vortexed and then incubated at 25 °C with continuous shaking at 200 rpm. For the comparison of bufalin, 3*-epi-*bufalin and 3*-oxo-*bufalin biotransformation, the incubation time was set at 24 h. For the time-dependent biotransformation of bufalin (containing the tissue homogenates), the incubation times were set at 0, 6, 12, 18, 24, and 36 h. After the incubation, 4 mL of dichloromethane (CH_2_Cl_2_) solution was added to terminate the biotransformation. The CH_2_Cl_2_ layer was separated, dried, and then redissolved in 500 μL of methanol. The solutions were subjected to HPLC analysis after passing through 0.22 μm microfiltration membranes.

### HPLC analysis

HPLC analysis was conducted using an Agilent 1200 series system controlled by Agilent ChemStation software. A 10 µL sample of the tissue incubation solution was injected and separated at 40 °C on a Phenomenex Luna C18 column (250 × 4.6 mm, 5 µm) with a flow rate of 1.0 mL/min. The elution gradient consisted of mobile phase A (0.1% formic acid in water) and mobile phase B (methanol) with the following profile: 0–10 min: 25–60% B, 10–40 min: 60–65% B, 40–50 min: 65–100% B. Detection of peaks was performed at 296 nm.

### Supplementary Information

**Additional file 1:** Instrument and equipment, biotransformation in toad kidney, and the NMR and MS spectra of 1-4.

## Data Availability

The data supporting the findings of this study were available on request from the corresponding author, upon reasonable request.
