# Supplementary material for: 3α-Hydroxybufadienolides in Bufo gallbladders: structural insights and biotransformation
Source: Nat Prod Bioprospect. 2024 Mar 4;14(1):19. doi: 10.1007/s13659-024-00442-2 (PMC10912398; doi:10.1007/s13659-024-00442-2)
Supplement: Supplementary file 1 — Additional file 1: Instrument and equipment, biotransformation in toad kidney, and the NMR and MS spectra of 1-4. [file 13659_2024_442_MOESM1_ESM.docx]

*Additional file*

**3α-Hydroxybufadienolides in Bufo Gallbladders: Structural Insights and Biotransformation**

Li-Jun Ruan ^1, 2^; Zhi-Jun Song^2^; Ren-Wang Jiang ^1*^

^1^ State Key Laboratory of Bioactive molecules and draggability assessment, College of Pharmacy, Jinan University, Guangzhou 510632, P. R. China.

^2^ National Engineering Research Center for Southwest Endangered Medicinal Materials Resources Development, Guangxi Botanical Garden of Medicinal Plants, Nanning 530023, P. R. China.

*** Correspondence:** trwjiang@jnu.edu.cn; Tel.: +8620-85221016 (R.W. Jiang)

# Instrument and equipment

Optical rotations were recorded in CH_3_OH on a Jasco P-1020 polarimeter at room temperature. UV spectra were determined in CH_3_OH on a Jasco V-550 UV/VIS spectrophotometer. IR spectra were obtained on a Jasco FR/IR-480 plus Fourier Transform infrared spectrometer using KBr pellets. Melting point was measured with an X-5 melting point apparatus without correction. X-ray diffraction study was carried out on an Agilent Gemini S Ultra Cu *Kα* radiation. HR-ESI-MS spectra were acquired on an Agilent 6210 ESI/TOF mass spectrometer. NMR spectra were measured on a Bruker AV-300 (300 and 75 MHz for ^1^H and ^13^C, respectively) spectrometer using solvent signals (CD_3_OD *δ*_H_ 3.310 / *δ*_C_ 49.00) as references. Column chromatographic methods were carried out on commercial silica gel (100-400 mesh, Qingdao Marine Chemical Plant, Qingdao, P. R. China). TLC analyses were carried out using pre-coated silica gel GF_254_ plates (Qingdao Marine Chemical Plant, Qingdao, P. R. China). Analytical high-performance liquid chromatography (HPLC) was carried out on an Agilent 1200 system chromatography equipped with Quatpump and DAD detector. HPLC-evaporative light scattering detection (ELSD) was used under Alltech ELSD 2000. GC-MS was determined on Finnigan Trace DSQ Single Quadrupole GC/MS (Thermo Electron Corporation) spectrometers. Semi-preparative HPLC (semi-RP-HPLC) was performed on a WUFENG LC-100 system equipped with a UV detector using a COSMOSIL Packed 5C_18_-MS-II column (5 μm, 250×10 mm). All solvents used in silica gel column and HPLC were of analytical grade (Shanghai Chemical Plant, Shanghai, P. R. China) and chromatographic grade (Fisher Scientific, undescribed Jersey, U. S. A), respectively.

# Biotransformation in toad kidney

Figure S1 The HPLC chromatogram of bufalin (B3), 3*-epi-*bufalin (αB3), and 3*-oxo-*bufalin (B3one) after incubated with toad kidney suspension for 24h: a and b, standards; c~d, the experimental group.

# NMR and MS spectra of 1-4

Figure S2 HR-ESI-MS spectrum of compound **1**

Figure S3 ^1^H NMR spectrum of compound **1** in CD_3_OD

Figure S4 ^13^C NMR spectrum of compound **1** in CD_3_OD

Figure S5 ^1^H -^1^H COSY spectrum of compound **1** in CD_3_OD

Figure S6 HSQC spectrum of compound **1** in CD_3_OD

Figure S7 HMBC spectrum of compound **1** in CD_3_OD

Figure S8 NOESY spectrum of compound **1** in CD_3_OD

Figure S9 HR-ESI-MS of compound **2**

Figure S10 ^1^H NMR spectrum of compound **2** in CD_3_OD

Figure S11 ^13^C NMR spectrum of compound **2** in CD_3_OD

Figure S12 ^1^H -^1^H COSY spectrum of compound **2** in CD_3_OD

Figure S13 HSQC spectrum of compound **2** in CD_3_OD

Figure S14 HMBC spectrum of compound **2** in CD_3_OD

Figure S15 NOESY spectrum of compound **2** in CD_3_OD

Figure S16 HR-ESI-MS of compound **3**

Figure S17 ^1^H NMR spectrum of compound **3** in CD_3_OD

Figure S18 13C NMR spectrum of compound 3 in CD3OD

Figure S19 HSQC spectrum of compound **3** in CD_3_OD

Figure S20 HMBC spectrum of compound **3** in CD_3_OD

Figure S21 HR-ESI-MS of compound **4**

Figure S22 ^1^H NMR spectrum of compound **4** in CD_3_OD

Figure S23 ^13^C NMR spectrum of compound **4** in CD_3_OD

Figure S24 HSQC spectrum of compound **4** in CD_3_OD

Figure S25 NOESY spectrum of compound **4** in CD_3_OD
